# Aticaprant (Clinically Developed Kappa-Opioid Receptor Antagonist) Combined With Naltrexone Prevents Alcohol “Relapse” Drinking

**DOI:** 10.13188/2327-204x.1000032

**Published:** 2022-05-16

**Authors:** Y Zhou, DC Zhou, MJ Kreek

**Affiliations:** Laboratory of the Biology of Addictive Diseases, The Rockefeller University, 1230 York Avenue, New York, NY 10065, USA

**Keywords:** Combination therapy, Aticaprant, KOR, Naltrexone, Alcohol deprivation effect, Relapse, Nor-BNI

## Abstract

Alcohol relapse is the treatment target for medications development for alcohol dependence. Aticaprant, a selective and short-acting kappa-opioid receptor (KOR) antagonist, has recently been under development for new clinical implications (depression or anhedonia). Recent studies have also found that aticaprant reduces alcohol intake and prevents stress- triggered alcohol seeking in rodents via a KOR-mediated mechanism. Here, we further investigated whether aticaprant alone or in combination with naltrexone (mu-opioid receptor [MOR] antagonist) altered alcohol relapse-like drinking using a mouse alcohol deprivation effect (ADE) paradigm to mimic the relapse episodes in human alcoholics. A long-acting and selective KOR antagonist nor-BNI was used as a reference compound for the effects of the KOR antagonism on the ADE. After 3-week intermittent-access alcohol drinking (two-bottle choice, 24-h access every other day), male and female mice displayed excessive alcohol intake and then pronounced ADE after 1-week abstinence. Aticaprant alone decreased alcohol ADE in a dose- dependent manner (1–3 mg/kg) in both males and females. Aticaprant at a lower dose (0.3 mg/kg) than the effective one (3 mg/kg) combined with a low dose of naltrexone (1 mg/kg) reduced the ADE in both sexes, and the combination was effective after a multi-dosing regimen (5 daily injections during the abstinence) without development of tolerance, suggesting synergistic effects of the combination. In contrast, nor-BNI alone or with naltrexone had no effect on the ADE in either sex. Our present study suggests that a combination of clinically developed, short-acting KOR antagonist aticaprant with low-dose naltrexone has therapeutic potential in alcohol “relapse” treatment.

## Introduction

It has been well established that classic KOR agonists induce alcohol-seeking behavior and promote alcohol relapse-like drinking [1–3]. Classic KOR agonists induce aversion, anxiety-like or depression-like behavior that may be responsible for alcohol seeking or relapse- like drinking or dysphoria in humans [4–7]. During acute alcohol withdrawal, pharmacological blockade of KOR attenuates alcohol seeking or drinking in rodents, as well as anxiety- or depression- like behaviors [8–16]. Withdrawal from alcohol activates KOR systems and induces negative mood states and symptoms, which is a part of negative reinforcing aspects of alcohol addiction [17,18].

After certain period of abstinence, a transient increase in alcohol intake is observed in both humans and rodents, which is characterized as the alcohol deprivation effect (ADE). The ADE has been widely used in rodent studies and been proved as suitable animal models for studying alcohol relapse [1,19,20]. Specifically, in our mouse model, after 1-week abstinence from 3-week intermittent-access alcohol drinking with excessive alcohol intake, both male and female mice show significant increases in alcohol consumption in the first 4 hours of alcohol access when alcohol is available again [21]. Our particularly relevant question was whether there was an upregulated KOR activity after prolonged withdrawal or abstinence and during alcohol relapse-like drinking, and if so, whether blockade of KOR could reduce the ADE or not. To date, however, there is no study investigating effects of the novel KOR antagonist aticaprant on alcohol “relapse” in rodent ADE models. Aticaprant (LY-2456302, CERC-501 or JNJ-67953964) is a selective and short-acting KOR antagonist and has recently been under development for the potential treatment of major depressive disorder [11,22,23]. Based on recent studies showing that aticaprant attenuates alcohol self-administration, reduces alcohol intake escalation, and prevents stress-induced seeking in rodents [11,12], we proposed a new hypothesis that aticaprant could prevent alcohol relapse-like drinking in ADE model. As alcohol relapse is an important target for medications development for alcoholism, we specifically investigated the pharmacological effects of aticaprant on mouse ADE in both sexes, to ascertain its potential as an anti-relapse compound. As nor-binaltorphimine (nor-BNI) is a selective, but long-acting, KOR antagonist, we purposely used nor-BNI as a reference compound and compared its effect with aticaprant.

Alcohol increases mu-opioid receptor (MOR)-mediated signal which is responsible for alcohol’s positive reinforcing and motivational properties and is highly involved in alcohol relapse episodes. MOR antagonist naltrexone decreases alcohol craving and relapse in humans [24], and relapse-like drinking in rodent ADE models [1,18]. Among multiple actions of alcohol in the CNS, both KOR and MOR activities are profoundly changed by alcohol exposure in humans [25,26], We further hypothesized that by targeting on both KOR and MOR, the combination of the clinically developed aticaprant and naltrexone would improve efficacy over the single-receptor approach on preventing relapse [18]. Therefore, the present study examined whether the appropriate combination of aticaprant and naltrexone could be more effective in preventing ADE than each alone.

## Materials & Methods

### Animals

Both male and female C57BL/6J mice (8-week-old) were purchased from The Jackson Laboratory (Bar Harbor, ME, USA), housed in a temperature-controlled (21 °C) facility, and acclimated on a 12-hour reverse light-dark cycle (lights off at 7:00 am) for at least one week prior to the experiments. The mice freely accessed to food and water and individually housed. All the animal care and experimental procedures were conducted according to Guide for Care and Use of Laboratory Animals (Institute of Laboratory Animal Resources Commission on Life Sciences 1996) and approved by the IACUC (Institutional Animal Care and Use Committee) of the Rockefeller University.

### Materials

Ethanol solution was prepared from 190 proof absolute ethyl alcohol (Pharmco- AAPER, Brookfield, CT, USA) and dissolved in tap water. Aticaprant was purchased from MedChemExpress, and suspended in 5% DMSO, 5% Cremophor and saline. Naltrexone hydrochloride from Sigma-Aldrich and nor-BNI from the NIDA Division of Drug Supply and Analytical Services were both dissolved in saline.

### Procedures

#### Alcohol deprivation effect (ADE) following intermittent-access alcohol drinking (Table S1).

Intermittent-access alcohol drinking model is a 2-bottle free choice paradigm with alcohol drinking every other day for 3 weeks as modified based on previous studies [21,27]. Both the water and alcohol (15%) solution sipper tubes were provided 3 hours after lights off, and the locations (left and right ones of the cage) of the bottles were randomly positioned to prevent from the development of side preference. The alcohol bottle filled with fresh 15% alcohol was kept for 24 h before being switched by the water bottle. Both water and alcohol intakes were recorded after 4, 8 and 24 hours of alcohol access in the drinking days and then calculated as consumed alcohol intake (g⁄kg) and relative preference for alcohol (alcohol intake ⁄ total fluid intake).

#### Injection of aticaprant or aticaprant plus naltrexone after excessive drinking.

After 3 weeks, alcohol (15%) was presented 30 min after a single injection of aticaprant (0.1, 0.3, 1 or 3 mg/kg, i.p.) or vehicle, and then alcohol and water intakes were recorded. The doses of aticaprant were based on recent publications [12]. The aticaprant plus naltrexone dose chosen was based on the above experiments and our recent studies [21,28], and mice were pretreated with aticaprant (0.3 mg/kg, i.p.) or vehicle 30 min before the test, followed by the second one of naltrexone (1 mg/kg) in saline10 min before the test.

**Both male and female mice were randomly assigned into the drug-**treated and vehicle groups with similar alcohol intake on day 21. On day 23, the experimenters [blinded to the drug codes] administered the drugs and vehicle before the test. In the vehicle control groups, the mice received one or two vehicles; and the mice in the drug groups received one (aticaprant) or two drugs (aticaprant plus naltrexone). Then, the alcohol and water bottles were presented, and their intakes were recorded. Of note, we did not observe significant sex differences with aticaprant or its combinations with naltrexone at the doses tested in our experiments, suggesting that the estrous cycle and associated hormones might not be important factors in the response to these treatments in females.

#### Injection of aticaprant alone or aticaprant plus naltrexone in ADE (Table S1).

On day 21, at the end of the 3-week intermittent-access alcohol drinking, 30% alcohol and water bottles were provided and their intakes at 4, 8 and 24 hours were recorded as the ones in the Baseline session. Then, the alcohol bottle was not offered for the following week. On day 28, after the 1-week abstinence, the alcohol (30%) bottle was provided once again 3 h after lights off and the alcohol and water intakes were recorded as the ones at 4, 8 and 24 h in the ADE session.

Both male and female mice were randomly assigned into the drug-treated and vehicle groups with similar alcohol intake in the Baseline session on day 21. On day 28, the experimenters who were blinded to the drug codes administered the drugs and vehicle before the ADE test. In the vehicle control groups, the mice received one or two vehicles; and the mice in the drug groups received one (aticaprant) or two drugs (aticaprant plus naltrexone). Then, the alcohol and water bottles were presented, and their intakes were recorded. (a) The doses of aticaprant were based on the above experiments in 3.2 section: mice received aticaprant (0.1 to 3 mg/kg, i.p.) or vehicle 30 min before the ADE test; (b) The doses of naltrexone were based on our recent study [28]: mice received naltrexone (0.3 or 1 mg/kg, i.p.) or vehicle 10 min before the ADE test; and (c) The aticaprant plus naltrexone dose was chosen after the above aticaprant alone and naltrexone alone experiments: mice received the first injection of aticaprant at low- dose (0.1 or 0.3 mg/kg) 30 min before the ADE test, followed by the second one of naltrexone (0.3 or 1 mg/kg) 10 min before the ADE test.

In the following experiment, the procedures were identical to the above experiment, with the following exceptions: mice received 5 consecutive injections of vehicles or aticaprant (0.3 mg/kg) plus naltrexone (1 mg/kg) during the 1-week abstinence. On the ADE test day, alcohol was presented 1 day after the last combination injection, and then alcohol and water intake values were recorded after 4, 8 and 24 hours in the ADE session.

#### Injection of nor-BNI alone or with naltrexone in ADE (Table S1).

Using the above same paradigm with an exception: the mice were pretreated with nor-BNI (30 mg/kg, i.p.) alone or vehicle on day 27 (1 day before the ADE test). The nor-BNI dose was based on our early publication in the intermittent-access drinking model [28, 29]. The nor-BNI plus naltrexone dose chosen was based on the above experiments, and mice were pretreated with nor-BNI (30 mg/kg, i.p.) or vehicle on day 27, followed by the second one of naltrexone (1 mg/kg) in saline 10 min before the drinking test on day 28.

#### Sucrose (caloric reinforcer) drinking.

The specificity of the action of aticaprant on alcohol intake was further examined on sucrose drinking behavior after the ADE at 3 mg/kg dose (the most effective dose tested on alcohol intake). The sucrose preference test in mice is sensitive to the function of brain rewarding systems and is widely used to measure the expression of anhedonia during alcohol abstinence [21] [30]. In these experiments, the ADE exposure was identical to the above one as described in section 3.1. During the 1-week abstinence, a sucrose solution (2%) was provided for 3 sessions with stable intakes. The mice were assigned to the vehicle- or aticaprant- treated groups with similar sucrose intakes before the test day. On the test day (after 1 week of alcohol abstinence), aticaprant (3 mg/kg) or vehicle was given 30 min before the sucrose tube was presented, and both sucrose and water intakes were recorded after 4, 8 and 24 hours of sucrose access.

### Data Analysis

Based on the levels of the mouse individual differences in our previous alcohol studies [21,28], power analyses were made to determine the number of mice required to reach statistical significances in the present studies. If similar effects of each treatment with no sex differences were found, data of each sex were analyzed separately. The group differences were analyzed using multiple-way ANOVAs for treatment (vehicle vs drug) and/or for sessions (Baseline vs ADE) in each sex, with a priori hypothesis that there were effects of ADE or drug treatment, based on the early publications and our new hypothesis in this study [1,19,21]. The multiple-way ANOVAs were followed by Newman-Keuls post-hoc tests, and the accepted significance was p<0.05 in Statistica 5.5 (StatSoft Inc, Tulsa, OK).

## Results

### Aticaprant alone

#### Excessive drinking.

**Figure 1:**
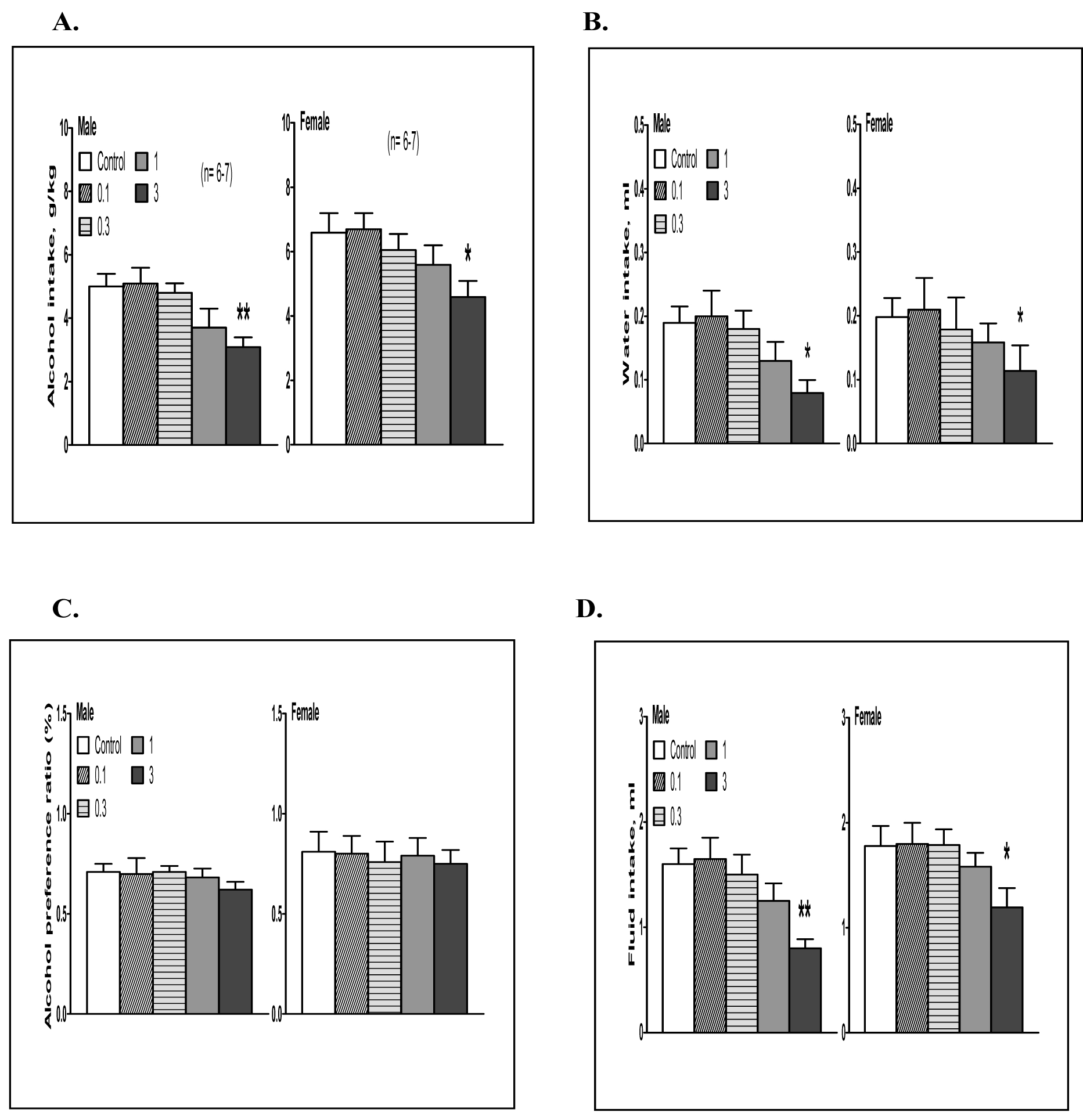
Effects of aticaprant (0.1–3 mg/kg) on alcohol intake (A), water intake (B), alcohol preference (C) and total fluid intake (D) at 4 hours in male (left) and female (right) mice after 3- week intermittent access alcohol drinking. *p<0.05 or **p<0.01 vs. control.

At the 4-hour time point, the dose responses of aticaprant (0.1, 0.3, 1 and 3 mg/kg) in terms of alcohol intake, water intake, alcohol preference and total fluid intake are presented in Figure 1.

##### A. Males:

For alcohol intake (Figure 1A, left), one-way ANOVA revealed a significant effect of aticaprant [F(4,28)=8.6, p<0.005], and post hoc analysis showed that in comparison with the vehicle group, the aticaprant-treated mice had less alcohol intake than the vehicle- treated ones at 3 mg/kg [p<0.01], with a marginal effect at 1 mg/kg [p=0.05]. For water intake (Figure 1B, left), one-way ANOVA showed a significant effect of aticaprant treatment [F(4,28)=5.2, p<0.01], and at 3 mg/kg, there were less water intake than the vehicle-treated control [p<0.05]. For preference ratio (Figure 1C, left), there were not any effect of aticaprant at any doses. For total fluid intake (Figure 1D, left), one-way ANOVA revealed a significant effect of aticaprant [F(4,28)=5.4, p<0.01], and post hoc analysis showed that in comparison with the vehicle group, the aticaprant-treated males at 3 mg/kg had less fluid intake [p<0.01].

##### B. Females:

For alcohol intake (Figure 1A, right), there was a significant effect of aticaprant [one-way ANOVA, F(4,28)=7.0, p<0.01], and there was a less alcohol intake than the vehicle-treated ones at 3 mg/kg [p<0.05]. For water intake (Figure 1B, right), there was a significant effect of aticaprant treatment [F(4,28)=4.2, p<0.01] and less water intake at 3 mg/kg than the control [p<0.05]. For preference ratio (Figure 1C, right), there was not any effect at any doses. For total fluid intake (Figure 1D, right), one-way ANOVA revealed a significant effect of aticaprant [F(4,28)=4.9, p<0.05], and the reduction at 3 mg/kg was significant [p<0.05].

Furthermore, after 8 or 24 hours or during the entire 24-hour drinking sessions, there were not any changes after aticaprant at any doses on either alcohol intake or water intake in either sex, as presented in Table 1 at 3 mg/kg of aticaprant.

#### ADE drinking.

**Figure 2:**
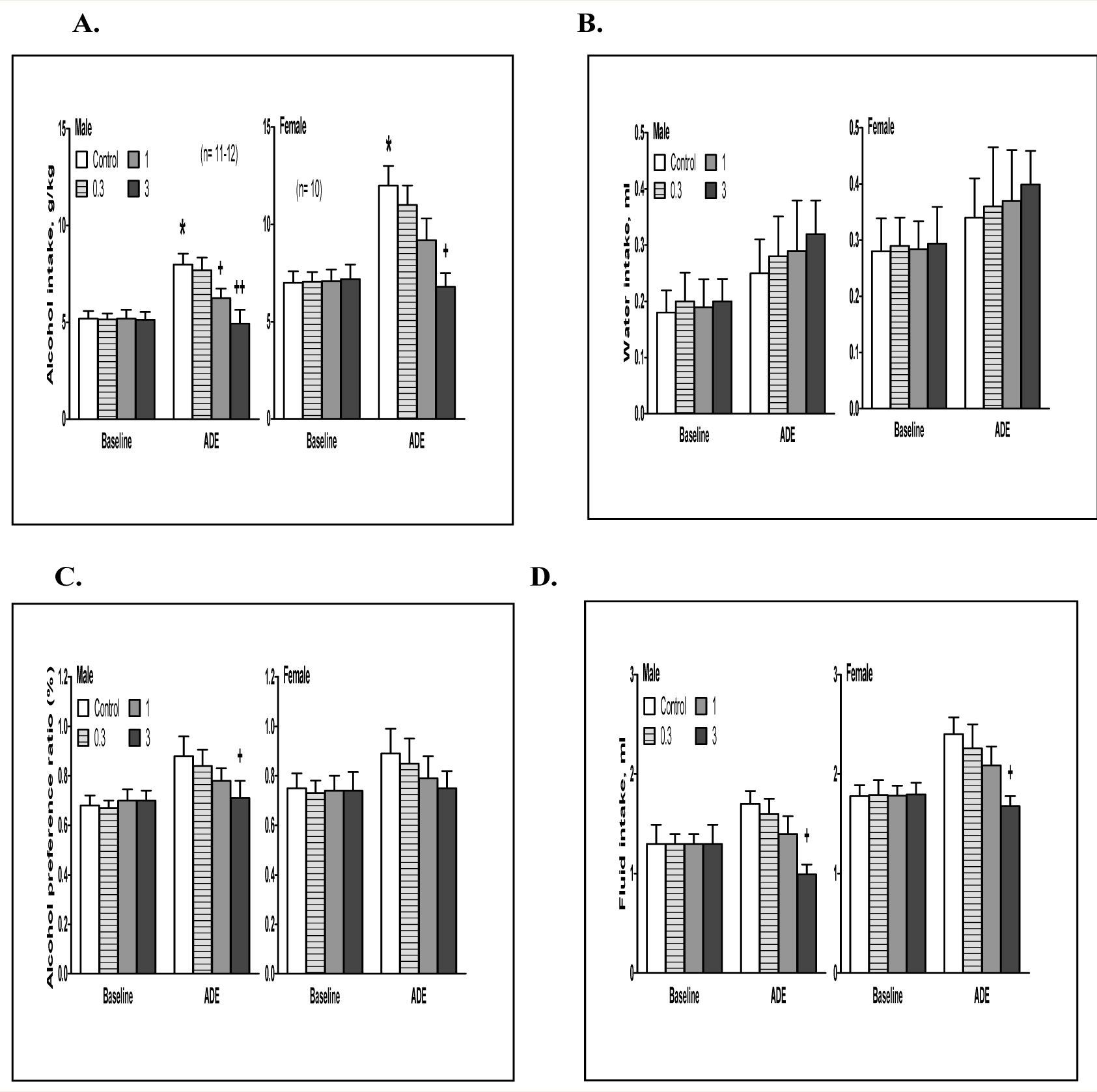
Effects of aticaprant (0.3–3 mg/kg) on alcohol intake (A), water intake (B), alcohol preference (C) and total fluid intake (D) at 4 hours in male (left) and female (right) mice after 1 week of abstinence from 3-week intermittent access alcohol drinking in alcohol deprivation effect (ADE) model. * p<0.05 vs. control baseline, and + p<0.05 or + + p<0.01 vs. control ADE.

In a pilot experiment, the effect of aticaprant at 0.1 mg/kg dose was tested in both males and females, and there was no difference between saline control and aticaprant treatment in either sex (data not shown). In the following experiment, three doses (0.3, 1 and 3 mg/kg) were tested, and the results on alcohol drinking at 4 hours are shown in Figure 2.

##### (A) Males.

For alcohol intake (Figure 2A, left), two-way ANOVA revealed significant effectts of aticaprant treatment [F(1,84)=3.8, p<0.05], session [F(1,84)=9.2, p<0.01], and interaction between session and treatment [F(1,84)=3.2, p<0.05]. Post hoc analysis showed that:

(1) there was more alcohol intake in the ADE session on day 28 than the baseline on day 21 [p<0.05]; and (2) at 1 mg/kg and 3 mg/kg, there were less alcohol intakes than the vehicle control in the ADE session [p<0.05 and p<0.01, respectively]. For water intake (Figure 2B, left), there was no effect of ADE or aticaprant at any doses. For alcohol preference, two-way ANOVA showed significant effects of aticaprant [F(1,84)=3.3, p<0.05], session [F(1,84)=5.1, p<0.05], and interaction between session and aticaprant treatment [F(1,84)=3.0, p<0.05]. Post hoc analysis showed that at 3 mg/kg, there was a less preference than the vehicle control in the ADE session [p<0.05]. For total fluid intake (Figure 2D, left), two-way ANOVA revealed significant effects of aticaprant [F(1,84)=4.3, p<0.05], session [F(1,84)=6.0, p<0.05], and interaction between session and aticaprant [F(1,84)=3.5, p<0.05], and that at 3 mg/kg, there was a less fluid intake than the vehicle control in the ADE session [p<0.05].

##### (B) Females.

For alcohol intake (Figure 2A, right), there were significant effects of aticaprant [two-way ANOVA, F(1,72)=3.3, p<0.05], session [F(1,72)=7.6, p<0.01], and interaction between session and aticaprant [F(1,72)=2.9, p<0.05]. There was more alcohol intake in the ADE session than the baseline [post-hoc test, p<0.05]; and the aticaprant-treated females at 3 mg/kg had less alcohol intake than the vehicle one in the ADE session [p<0.05], with a marginally significant decrease at 1 mg/kg [p=0.07]. For water intake (Figure 2B, right), there was not any effect of ADE or aticaprant at any doses. For alcohol preference, there were significant effects of aticaprant [two-way ANOVA, F(1,72)=2.9, p<0.05] and session [F(1,72)=4.6, p<0.05], and the aticaprant-treated females at 3 mg/kg had a slight less preference than the vehicle one in the ADE session [p=0.07] (Figure 2C, right). For total fluid intake (Figure 2D, left), two-way ANOVA revealed significant effects of aticaprant [F(1,72)=3.3, p<0.05], session [F(1,72)=4.2, p<0.05], and interaction between session and aticaprant [F(1,72)=3.0, p<0.05]. At 3 mg/kg, there was a less fluid intake than the vehicle control in the ADE session [p<0.05].

After 8 or 24 hours, there was not any change after aticaprant at any doses on either alcohol or water intake in either sex, as presented in Table 2 at 3 mg/kg of aticaprant.

#### Sucrose drinking (intake and preference after 1-week alcohol abstinence).

As both alcohol and sucrose are caloric reinforcers, the specificity of the aticaprant effect on alcohol intake was examined after aticaprant injection at 3 mg/kg on sucrose drinking after 1 week of alcohol abstinence. As shown in Table 3, there was no effect of 3 mg/kg aticaprant on 2% sucrose intake in either males or females after 4 hours. For water intake, however, the aticaprant- treated mice had a less water intake than the control mice in both males [Student’s t-test (1,15)=5.4, p<0.05] and females [Student’s t-test (1,15)=4.9, p<0.05]. For preference ratio, the aticaprant-treated mice had more preferences than the control mice in both male [Student’s t-test (1,15)=5.9, p<0.05] and female mice [Student’s t-test (1, 15)=5.1, p<0.05]. For total fluid intake, there were no effects in either sex. After 8 or 24 hours, there was not any change by aticaprant on either sucrose intake or water intake in either sex (data not shown).

### Aticaprant plus naltrexone

#### Excessive drinking.

In a pilot study, we tested the effect of 0.1 mg/kg aticaprant combined with 0.3 mg/kg naltrexone and found that the combination did not have a significant effect on excessive drinking in either sex (data not shown). With higher doses of both compounds: aticaprant at 0.3 mg/kg and naltrexone at 1mg/kg, there were significant effects after 4 hours of alcohol drinking (Table 4): For alcohol intake, two-way ANOVA revealed a significant effect of the combination treatment [F(1,27)=8.9, p<0.01], and the combination significantly reduced alcohol intake in both males and females [p<0.05 for both]. For preference ratio, two-way ANOVA also revealed a significant effect of the treatment [F(1,27)=10.1, p<0.01], and the combination significantly reduced preference ratio in both sexes [p<0.05 for both]. For water intake or total fluid intake, there were not any changes by the combination in either sex. After 8 or 24 hours, there were no significant effects on alcohol intake in either sex (data not shown).

#### ADE drinking.

**Figure 3:**
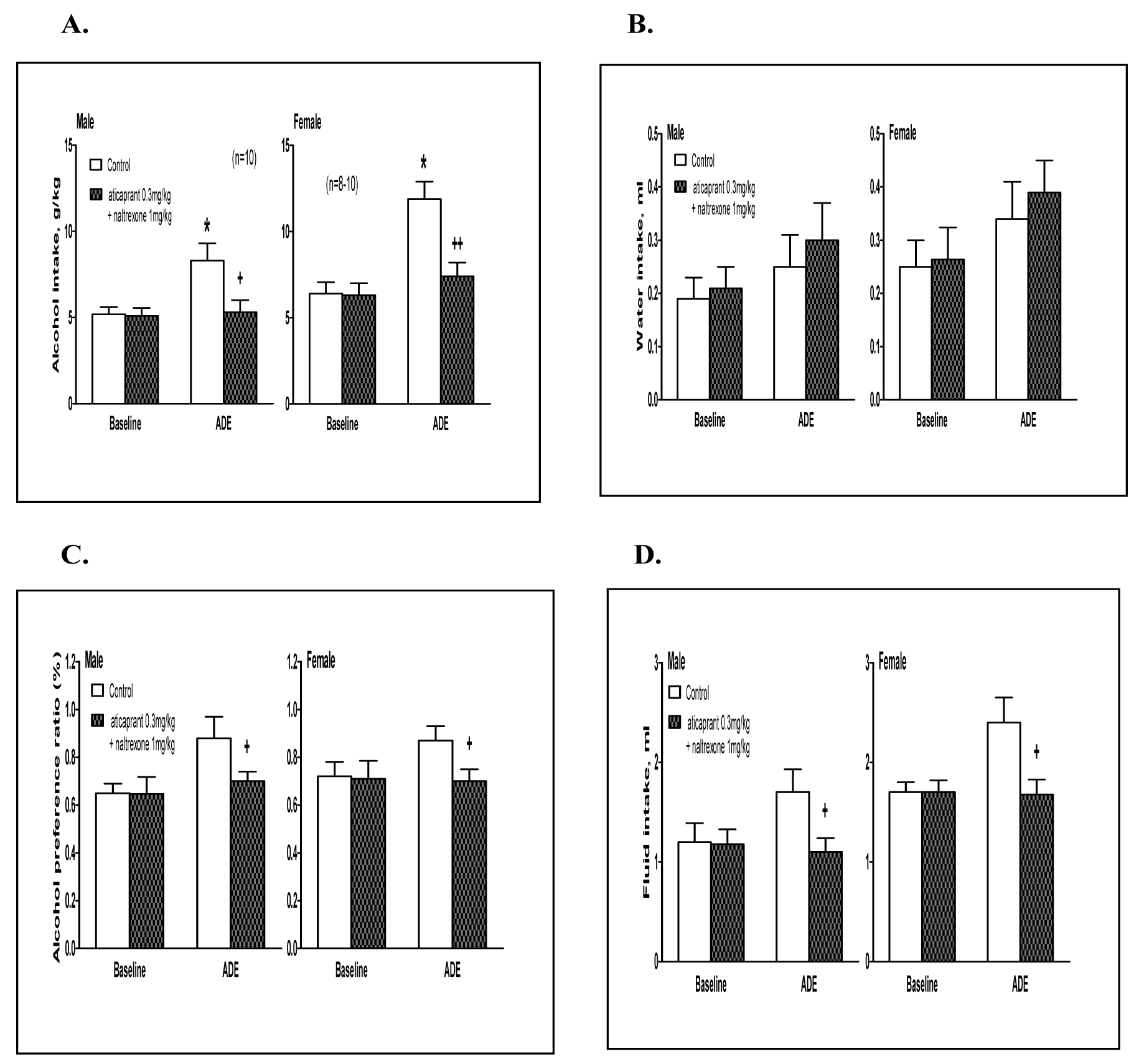
Effects of aticaprant (0.3 mg/kg) combined with naltrexone (1 mg/kg) on alcohol intake (A), water intake (B), alcohol preference (C) and total fluid intake (D) at 4 hours in male (left) and female (right) mice after 1 week of abstinence from 3-week intermittent-access alcohol drinking in alcohol deprivation effect (ADE) model. * p<0.05 vs. control baseline, and + p<0.05 or ++ p<0.01 vs. control ADE.

In a pilot study, we also tested the combination of 0.1 mg/kg aticaprant with naltrexone at 0.3 mg/kg, and there was no effect on ADE at 4 hours in either sex (Table 5). With higher doses: aticaprant at 0.3 mg/kg and naltrexone at 1 mg/kg, the ADE alcohol intake at 4 hours is reduced as shown in Figure 3.

##### (A) Males.

For alcohol intake (Figure 3A, left), two-way ANOVA revealed significant effects of treatment [F(1,36)=4.9, p<0.05], session [F(1,36)=5.2, p<0.05] and their interaction [F(1,36)=3.6, p<0.05]. The post-hoc results showed that males had more intake in the ADE session than that in the baseline [p<0.05]. However, the aticaprant plus naltrexone-treated males had less intake than the vehicle controls in the ADE session [p<0.05]. For water intake, there was no effect of ADE or the combination (Figure 3B, left). For alcohol preference, there was a significant effect of treatment × session interaction [two-way ANOVA, F(1,36)=4.4, p<0.05], and the males treated with aticaprant plus naltrexone had a less preference than the vehicle ones in the ADE session [p<0.05] (Figure 3C, left). For total fluid intake (Figure 3D, left), two-way ANOVA revealed significant effects of treatment [F(1,36)=3.4, p<0.05] and treatment × session interaction [F(1,36)=3.2, p<0.05]. In the males treated with the combination, there was a less fluid intake than the vehicle-treated control in the ADE session [p<0.05].

##### (B) Females.

For alcohol intake (Figure 3A, right), two-way ANOVA showed significant effects of treatment [F(1,32)=6.9, p<0.05], session [F(1,32)=8.6, p<0.01] and their interaction [F(1,32)=4.5, p<0.05]. Females had more intake in the ADE session than that in the baseline [p<0.05]; however, the aticaprant plus naltrexone-treated females had a less intake than the vehicle controls in the ADE session [p<0.01]. For water intake, there was no effect of ADE or the combination (Figure 3B, right). For alcohol preference, there was a significant effect of treatment and session interaction [F(1,32)=4.8, p<0.05], and the females treated with aticaprant plus naltrexone had a less preference in the ADE session than the vehicle ones [p<0.05] (Figure 3C, right). For total fluid intake (Figure 3D, right), two-way ANOVA revealed significant effects of treatment [F(1,32)=3.6, p<0.05], and treatment × session interaction [F(1,32)=3.5, p<0.05]. The females treated with the combination had less fluid intake than the vehicle-treated control in the ADE session [p<0.05].

After 8 or 24 hours, there were no changes by the combination on alcohol intake or water intake in either sex, as presented in Table 6.

#### Sucrose drinking.

The specificity of the combination of aticaprant with naltrexone on alcohol drinking was further examined on sucrose drinking after 1 week of alcohol abstinence. There was no effect of the combination on 2% sucrose intake in either sex after 4 hours (Table 7) or after 8 or 24 hours (data not shown).

#### Repeated combination on ADE drinking.

In this experiment, we tested the effect of the repeated combination administrations in both males and females and found similar reducing effects on the ADE. As there were no significant sex differences again, the data of each sex are presented separately (Table 8): [A] In males at 4 hours (Table 8A), two-way ANOVA showed a significant effect of the combination treatment [F(1,20)=5.6, p<0.05], and that the combination- treated males had a less intake than the vehicle-treated ones in the ADE session [p<0.05]. To test our a priori hypothesis that there was an ADE, the post-hoc result was included here: the vehicle-treated males had a significant ADE [p<0.05] (Table 8A), though 2-way ANOVA did not reveal any significant effect of the ADE session; [B] In females at 4 hours (Table 8B), two- way ANOVA revealed a significant effect of session [F(1,26)=11, p<0.001] and post hoc analysis showed that the females had more intake in the ADE session than the baseline [p<0.01], and the combination-treated females did not show the ADE [p<0.05]; and [C] However, there was no significant effect of the repeated combination treatment on either males or females after 8 or 24 hours (data not shown).

### nor-BNI alone or with naltrexone

The results with 30 mg/kg nor-BNI alone at 4 hours are shown in Table 9, or with 1 mg/kg naltrexone in Table 10. There was not any effect on ADE in either sex at 4 hours. After 8 or 24 hours, there were no changes by either nor-BNI alone or with naltrexone (data not shown).

## Discussion

In several recent studies with different alcohol self-administration models, the selective and short-acting KOR antagonist aticaprant has been found to reduce alcohol intake and prevents stress-induced alcohol seeking [11,12]. To further examine whether aticaprant could have potential as an anti-relapse compound, the present study utilized a mouse ADE paradigm to mimic alcohol relapse in human alcoholics and explored the potential of aticaprant in preventing relapse-like drinking after alcohol abstinence from excessive intake. The ADE has been studied as a rodent model of craving for alcohol and alcohol relapse drinking with good predictive validity [20]. Specifically, we tested aticaprant with 0.1–3 mg/kg doses and found that aticaprant at 3 mg/kg significantly reduced alcohol intake in both the ADE relapse-like model and excessive drinking model after 4 hours in both males and females. The effect of aticaprant on the mouse ADE intake may be not due to its general inhibition of consumption behaviors, as paticaprant at the effective dose 3 mg/kg did not change sucrose intake. Therefore, aticaprant may provide potential treatments for alcohol relapse. Our finding would constitute additional information of the aticaprant properties in alcohol, nicotine, or opiate -related studies [11,12,31,32].

Increased KOR activity might occur after alcohol exposure or during different phases of alcohol withdrawal and KOR antagonists might be useful during withdrawal or abstinence [3,10,12,26,33–41]. Recent human PET study found that compared to healthy controls, alcohol-dependent subjects have altered KOR availability across multiple brain regions, including the frontal cortex, dorsal striatum, and amygdala [25,26]. Our new study here suggests that KOR activity is involved in relapse-like drinking, as pharmacological blockade of KOR using the short-acting antagonist aticaprant prevented mouse ADE, though more study is needed.

Previous work has found that KOR antagonists do *not* alter sucrose drinking in stress- naïve animals but increases sucrose preference in stress exposed animals due to “anti- depression” properties [13,15]. Though aticaprant does not produce any reward in rodents [14], repeated administration of aticaprant has been found to correct the reduction in sucrose preference or intake after chronic stress and is under development for the potential treatment of major depressive disorder [15,23]. Consistently, our results here showed that aticaprant at 3 mg/kg (the most effective dose for reducing alcohol intake) increased sucrose preference in mice after experiencing repeated stress during 3-week intermittent alcohol abstinence and after 1-week abstinence, suggesting that the blockade of KOR activity by aticaprant could modulate sensitivity to sucrose reward during the alcohol abstinence. Alternatively, aticaprant may increase preference for more palatable reinforcers, such as sucrose, with decreased preferences for less palatable reinforcers (15–30% alcohol), though aticaprant itself is not rewarding. However, we did observe that aticaprant decreased water intake in the excessive alcohol drinking experiment, suggesting that aticaprant may have decreased alcohol intake through off-target effects, such as general motivation to consume any fluid. Therefore, this question led us to specifically examine its effect on sucrose drinking behavior and we found that aticaprant did not change sucrose intake, with water intake reduction, arguing that the reductions of alcohol drinking could *not* be attributed to general inhibition of consumption behaviors. As reported by other groups, aticaprant as a selective and short-acting KOR antagonist, does not display any sedative activity or cause “depression or dysphoria” in rodents as found with classic KOP-r agonists and has recently been under development for new clinical implications as anti- depressant [11,15,23]. It is unclear why aticaprant decreased water intake in some of our experiments.

It was noteworthy that in contrast to aticaprant, the long-acting KOR antagonist nor-BNI at 30 mg/kg did not prevent the ADE in either sex. In our previous dose-response experiments, 5–20 mg/kg of nor-BNI was tested and all failed to show any effect of the long-acting KOR antagonist on mouse relapse-like drinking [28]. Consistently, an early study by another group reported that nor-BNI at 10 mg/kg did not alter the ADE in male rats with long-term (one and half years) of alcohol drinking experience in a 4-bottle choice drinking paradigm [19]. Using an operant alcohol self-administration paradigm, nor-BNI had no effect on the rat ADE or alcohol intake neither [19,42]. Nevertheless, the behavioral observations explored in both the rat and mouse studies with nor-BNI suggest that the long-acting KOR antagonist may represent different actions and mechanisms from short-acting antagonists [4,22,43].

In the second main objective of our present study, we provide clear experimental evidence showing that the combination of aticaprant with MOR antagonist naltrexone is more effective than either single drug alone. When aticaprant (0.3 mg/kg) with naltrexone (1 mg/kg) together was tested in both the excessive drinking model and ADE relapse-like models, there was a profound and synergistic effect of the combination on decreasing excessive intake and preventing ADE in both males and females, as each compound at its low dose did not have any effect alone. The combination did not alter sucrose intake or water intake, indicating an alcohol- specific effect of the combination. However, one possible concern about the repeated use of aticaprant is that its KOR antagonistic activity after repeated administration could result in the development of tolerance. Therefore, our study purposely examined the efficacy of a multiple- dosing regimen (5 daily injections before the ADE during the 1-week abstinence) to mimic the multiple-dosing treatment in the clinic. Like the effect of single administration of the combination (0.3 mg/kg aticaprant with 1 mg/kg naltrexone), the repeated pretreatments of the combination during the abstinence effectively prevented the ADE drinking in both sexes, and the 1-week daily administrations did not show any development of tolerance in our multiple-dosing regimen, consistent with the human clinical testing and preclinical studies [15,23,44]. As the single-receptor pharmacotherapies have been recently found to have modest therapeutic value on alcohol relapse, there is an obvious need for better efficacy [18,20], and our new finding here has provided promising *in vivo* data demonstrating that the clinically developed short-acting KOR antagonist aticaprant, in combination with low-dose naltrexone, may offer a novel strategy to treat alcohol relapse.

## Summary

Alcoholism remains a big public health problem with limited treatment choices. The KOR system has been a potential target for the development of drug addiction treatments, as many preclinical studies consistently demonstrated that KOR antagonists decrease drug- taking and seeking behaviors, as well as drug withdrawal-related behaviors in rodents, especially under stress conditions [17]. Consistently, we found that aticaprant (a selective and short-acting KOR antagonist) alone decreased alcohol intake and prevented “relapse” drinking in both the excessive alcohol drinking and alcohol relapse-like (ADE) mouse models respectively, with a paralleled reduction of water intake. However, several recent clinical reports have found negative results on drug addiction, showing that aticaprant treatment is ineffective on nicotine or cocaine (for example, [32]), though aticaprant has been under development for the potential treatment of major depressive disorder in humans [23]. Therefore, more effective treatment strategies need to be further developed with the KOR antagonist in the preclinical research. As both KOR and MOR systems interact each other in neurobiological processes of drug addiction, the present study specifically tested whether the effectiveness of aticaprant can be improved under MOR antagonism by naltrexone. Of interest, when aticaprant co-administered with naltrexone together, aticaprant at sub-effective doses profoundly reduced excessive alcohol consumption and prevented relapse-like drinking, without affecting sucrose or water intake, particularly with a multiple-dosing regimen of the combination. Together, our new study strongly suggests that aticaprant in combination with naltrexone offers a novel approach for alcoholism treatment.

## Supplementary Material

2

## Figures and Tables

**Table 1:** No effect of aticaprant (3 mg/kg) at 8 or 24 hours on alcohol intake or water intake in an excessive alcohol drinking model in male (A, n=6–7) and female (B, n=6–7) mice after 3-week intermittent-access alcohol drinking. See Figure 1 at 4-hour time point.

**A. Male**			
	Time point	vehicle	3 mg/kg
Alcohol,	5–8 hour	3.3 ± 0.6	3.8 ± 1.2
g/kg	9–24 hour	10.7 ± 1.4	13.1 ± 1.9
Water,	5–8 hour	0.25 ± 0.07	0.31 ± 0.09
ml	9–24 hour	0.45 ± 0.09	0.60 ± 0.12
**B. Female**			
	Time point	vehicle	3 mg/kg
Alcohol,	5–8 hour	4.7 ± 1.0	5.1 ± 1.1
g/kg	9–24 hour	13.0 ± 1.0	15.7 ± 1.6
Water,	5–8 hour	0.33 ± 0.07	0.39 ± 0.11
ml	9–24 hour	0.54 ± 0.04	0.61 ± 0.12

**Table 2:** No effect of aticaprant (3 mg/kg) at 8 or 24 hours on alcohol intake (A, B) or water intake (C, D) in an alcohol deprivation effect (ADE) model in male (A and C, n=11–12) and female (B and D, n=10) mice after 1 week of abstinence from 3-week intermittent-access alcohol drinking. See Figure 2 at 4-hour time point.

**A. Male**					
		Vehicle		3 mg/kg	aticaprant
	Time point	Baseline	ADE	Baseline	ADE
Alcohol,	5–8 hour	3.0 ± 0.4	4.0 ± 1.1	3.2 ± 0.4	3.5 ± 1.2
g/kg	9–24 hour	7.0 ± 1.1	8.1 ± 2.4	7.1 ± 1.5	7.9 ± 2.8
**B. Female**					
		Vehicle		3 mg/kg	aticaprant
	Time point	Baseline	ADE	Baseline	ADE
Alcohol,	5–8 hour	6.1 ± 0.6	7.0 ± 2.1	6.2 ± 0.5	7.0 ± 1.5
g/kg	9–24 hour	9.6 ± 2.0	12 ± 2.0	9.9 ± 1.9	11 ± 2.2
**A. Male**					
		Vehicle		3 mg/kg	aticaprant
	Time point	Baseline	ADE	Baseline	ADE
Water,	5–8 hour	0.48 ± 0.16	0.54 ± 0.23	0.49 ± 0.14	0.55 ± 0.29
ml	9–24 hour	0.83 ± 0.12	0.89 ± 0.19	0.84 ± 0.15	0.94 ± 0.28
**B. Female**					
		Vehicle		3 mg/kg	aticaprant
	Time point	Baseline	ADE	Baseline	ADE
Water,	5–8 hour	0.53 ± 0.15	0.64 ± 0.21	0.54 ± 0.08	0.59 ± 0.27
ml	9–24 hour	1.2 ± 0.31	1.4 ± 0.25	1.1 ± 0.33	1.5 ± 0.29

**Table 3:** Effects of aticaprant (3 mg/kg) on sucrose (2%) intake, water intake and sucrose preference at 4 hours in male (A, n=8) and female (B, n=8) mice after 1 week of abstinence from 3-week intermittent-access alcohol drinking.

**A. Male**		
	Vehicle	Aticaprant
Sucrose, g/kg	1.8 ± 0.09	1.9 ± 0.11
Water, ml	0.21 ± 0.03	0.10 ± 0.02*
Preference ratio	0.89 ± 0.02	0.97 ± 0.03*
Total fluid intake, ml	2.4 ± 0.4	2.1 ± 0.3
**B. Female**		
	Vehicle	Aticaprant
Sucrose, g/kg	2.1 ± 0.13	2.3 ± 0.11
Water, ml	0.23 ± 0.04	0.14 ± 0.02*
Preference ratio	0.91 ± 0.03	0.97 ± 0.02*
Total fluid intake, ml	2.7 ± 0.5	2.4 ± 0.4

* p<0.05 vs vehicle control.

**Table 4:** Effects of aticaprant at 0.3 mg/kg combined with naltrexone (1 mg/kg) on alcohol intake, water intake, alcohol preference and total fluid intake at 4 hours in an excessive alcohol drinking model in male (n=7–8) and female (n=5–7) mice after 3-week intermittent-access alcohol drinking.

	Male		Female	
Treatments	Vehicle+Vehicle	Aticaprant + naltrexone	Vehicle+Vehicle	Aticaprant+naltrexone
Alcohol, g/kg	4.7 ± 0.6	3.1 ± 1.0*	5.2 ± 0.8	3.8 ± 0.9*
Water, ml	0.22 ± 0.09	0.25 ± 0.13	0.24 ± 0.15	0.28 ± 0.19
Preference ratio	0.76 ± 0.06	0.54 ± 0.06*	0.83 ± 0.07	0.65 ± 0.09*
Total fluid intake, ml	1.9 ± 0.4	1.6 ± 0.7	2.3 ± 0.7	2.0 ± 0.9

* p<0.05 vs vehicle control.

**Table 5:** No effect of aticaprant (0.1 mg/kg) combined with naltrexone (0.3 mg/kg) on alcohol intake in an alcohol deprivation effect (ADE) model at 4 hours in male (A, n=6) and female (B, n=6) mice after 1 week of abstinence from 3-week intermittent-access alcohol drinking. In the males at 4 hours (**A**), two-way ANOVA revealed a significant effect of session only [F (1,33)=6.4, p<0.01], and the males had more intake in the ADE session than that in the baseline [p<0.05]. In the females at 4 hours (**B**), two-way ANOVA revealed a significant effect of session only [F(1,33)=7.5, p<0.01], and the females had more intake in the ADE session than that in the baseline [p<0.05].

**A. Male**				
	Vehicle +	Vehicle	0.1 mg/kg + 0.3 mg/kg	aticaprant naltrexone
	Baseline	ADE	Baseline	ADE
alcohol, g/kg	5.1 ± 0.6	7.1 ± 1.0*	5.0 ± 0.5	6.8 ± 1.0*
Water, ml	0.25 ± 0.09	0.29 ± 0.11	0.24 ± 0.10	0.29 ± 0.12
**B. Female**				
	Vehicle +	Vehicle	0.1 mg/kg + 0.3 mg/kg	aticaprant naltrexone
	Baseline	ADE	Baseline	ADE
alcohol, g/kg	6.2 ± 0.8	11.1 ± 1.3*	6.1± 0.7	10.3 ± 1.1*
Water, ml	0.33 ± 0.10	0.38 ± 0.20	0.32 ± 0.11	0.39 ± 0.12

* p<0.05 vs control baseline.

**Table 6:** No effect of aticaprant (0.3 mg/kg) combined with naltrexone (1 mg/kg) at 8 or 24 hours on alcohol intake (A, B) or water intake (C, D) in an alcohol deprivation effect (ADE) model in male (n=10) and female (n=8–10) mice after 1 week of abstinence from 3-week intermittent- access alcohol drinking. See Figure 3 at 4-hour time point.

**A. Male**					
		Vehicle		aticaprant + naltrexone	
	Time point	Baseline	ADE	Baseline	ADE
Alcohol,	5–8 hour	3.2 ± 0.5	4.0 ± 1.1	3.1 ± 0.6	3.5 ± 1.2
g/kg	9–24 hour	7.2 ± 1.5	7.7 ± 2.7	7.1 ± 1.3	7.8 ± 2.5
**B. Female**					
		Vehicle		aticaprant + naltrexone	
	Time point	Baseline	ADE	Baseline	ADE
Alcohol,	5–8 hour	5.9 ± 0.77	7.2 ± 0.90	6.0 ± 0.59	7.0 ± 0.90
g/kg	9–24 hour	8.0 ± 1.0	10.1 ± 2.1	8.1 ± 1.5	9.8 ± 2.0
**A. Male**					
		Vehicle		aticaprant + naltrexone	
	Time point	Baseline	ADE	Baseline	ADE
Water,	5–8 hour	0.41 ± 0.13	0.50 ± 0.18	0.42 ± 0.15	0.53 ± 0.15
ml	9–24 hour	0.80 ± 0.10	0.88 ± 0.15	0.82 ± 0.14	0.90 ± 0.21
**B. Female**					
		Vehicle		aticaprant + naltrexone	
	Time point	Baseline	ADE	Baseline	ADE
Water,	5–8 hour	0.50 ± 0.15	0.60 ± 0.17	0.51 ± 0.13	0.66 ± 0.22
ml	9–24 hour	1.1 ± 0.22	1.2 ± 0.25	1.0 ± 0.19	1.3 ± 0.29

**Table 7:** No effect of aticaprant (0.3 mg/kg) combined with naltrexone (1 mg/kg) on sucrose (2%) intake or water intake at 4 hours in male (A, n=9) and female (B, n=7–8) mice after 1 week of abstinence from 3-week intermittent-access alcohol drinking.

**A. Male**		
	Vehicle	Aticaprant + naltrexone
Sucrose, g/kg	1.9 ± 0.09	1.7 ± 0.08
Water, ml	0.23 ± 0.04	0.20 ± 0.04
**B. Female**		
	Vehicle	Aticaprant + naltrexone
Sucrose, g/kg	2.3 ± 0.11	2.1 ± 0.09
Water, ml	0.25 ± 0.05	0.24 ± 0.04

**Table 8:** Effects of five repeated administration of aticaprant (0.3 mg/kg) combined with naltrexone (1 mg/kg) on alcohol intake, water intake, alcohol preference and total fluid intake at 4 hours in male (A, n=6) and female (B, n=7–8) mice after 1 week of abstinence from 3-week intermittent-access alcohol drinking in alcohol deprivation effect (ADE) model.

**A. Male**				
	Vehicle +	Vehicle	0.3 mg/kg + 1 mg/kg	aticaprant naltrexone
	Baseline	ADE	Baseline	ADE
alcohol, g/kg	5.8 ± 0.5	7.5 ± 0.7*	5.4 ± 0.5	5.8 ± 0.6
Water, ml	0.22 ± 0.05	0.25 ± 0.07	0.24 ± 0.07	0.28 ± 0.10
Preference ratio	0.65 ± 0.05	0.72 ± 0.08	0.66 ± 0.06	0.68 ± 0.08
Total fluid, ml	1.3 ± 0.3	1.7 ± 0.4	1.2 ± 0.4	1.4 ± 0.6
**B. Female**				
	Vehicle +	Vehicle	0.3 mg/kg + 1 mg/kg	aticaprant naltrexone
	Baseline	ADE	Baseline	ADE
alcohol, g/kg	6.4 ± 0.7	12.6 ± 1.3**	7.0± 1.1	8.3 ± 1.1 +
Water, ml	0.26 ± 0.08	0.31 ± 0.09	0.28 ± 0.10	0.33 ± 0.09
Preference ratio	0.69 ± 0.06	0.83 ± 0.08	0.70 ± 0.07	0.75 ± 0.11
Total fluid, ml	1.6 ± 0.6	2.2 ± 0.7	1.6 ± 0.7	1.8 ± 0.8

* p<0.05 or

** p<0.01 vs. control baseline, and

+ p<0.05 vs control ADE.

**Table 9:** No effects of nor-BNI (30 mg/kg) on alcohol intake in an alcohol deprivation effect (ADE) model at 4 hours in male (A, n=9) and female (B, n=9) mice after 1 week of abstinence from 3-week intermittent-access alcohol drinking. **A**. Two-way ANOVA revealed a significant effect of session only [F(1,32)=8.1, p<0.01], and ADE session had more alcohol intake than baseline [p<0.05]. **B**. Two-way ANOVA revealed a significant effect of session only [F (1,32)=8.6, p<0.01], and ADE session had more alcohol intake than baseline [p<0.05].

**A. Male**				
	Vehicle		nor-BNI	alone
	Baseline	ADE	Baseline	ADE
Alcohol, g/kg	4.7 ± 0.7	7.4 ± 0.9*	5.0 ± 0.7	7.3 ± 1.1*
Water, ml	0.23 ± 0.09	0.27 ± 0.15	0.22 ± 0.14	0.25 ± 0.11
**B. Female**				
	Vehicle		nor-BNI	alone
	Baseline	ADE	Baseline	ADE
Alcohol, g/kg	6.5 ± 0.5	12.0 ± 1.9*	6.5 ± 0.6	11.7 ± 1.2*
Water, ml	0.28 ± 0.17	0.32 ± 0.22	0.30 ± 0.14	0.34 ± 0.15

* p<0.05 vs control Baseline.

**Table 10:** No effects of nor-BNI (30 mg/kg) with naltrexone (1 mg/kg) on alcohol intake in an alcohol deprivation effect (ADE) model at 4 hours in male (A, n=8–12) and female (B, n=10) mice after 1 week of abstinence from 3-week intermittent-access alcohol drinking. **A**. Two-wayANOVA revealed a significant effect of session only [F(1,36)=6.1, p<0.05], and ADE session had more alcohol intake than baseline [p<0.05]. **B**. Two-way ANOVA revealed a significant effect of session only [F (1,36)=7.4, p<0.01], and ADE session had more alcohol intake than baseline [p<0.05].

**A. Male**				
	Vehicle		nor-BNI +	naltrexone
	Baseline	ADE	Baseline	ADE
Alcohol, g/kg	4.2 ± 0.5	6.4 ± 1.1*	4.0 ± 0.7	6.3 ± 1.7*
Water, ml	0.20 ± 0.09	0.24 ± 0.12	0.21 ± 0.15	0.25 ± 0.13
**B. Female**				
	Vehicle		nor-BNI +	naltrexone
	Baseline	ADE	Baseline	ADE
Alcohol, g/kg	6.1 ± 0.8	10.0 ± 1.8*	6.0 ± 0.8	11 ± 1.7*
Water, ml	0.29 ± 0.15	0.36 ± 0.21	0.30 ± 0.16	0.37 ± 0.17

* p<0.05 vs control Baseline.
