# Supplementary material for: Aticaprant (Clinically Developed Kappa-Opioid Receptor Antagonist) Combined With Naltrexone Prevents Alcohol “Relapse” Drinking
Source: J Pharm Pharmacol (Los Angel). Author manuscript; Available in PMC 2022 Jul 12. (PMC9275124; doi:10.13188/2327-204x.1000032)
Supplement: 2 [file NIHMS1810760-supplement-2.pdf]

Table S1. Experimental timelines. The 3-week intermittent access alcohol drinking (15% alcohol vs. water) and after1-week abstinence (alcohol deprivation effect, ADE) with aticaprant, naltrexone, nor-BNI or their combinations.

A.

Weeks 1-3	Day 23
Intermittent access in two-bottle choice model (15% alcohol vs. water) with 24-h access every other day	Aticaprant alone or with naltrexone<br><br>drinking test

B.

Weeks 1-3	Week 4	Day 27 or 28
Intermittent access in a two-bottle choice model (15% alcohol vs. water) with 24-h access every other day	1-week abstinence	Nor-BNI or Aticaprant alone or with naltrexone, ADE test

C.

Weeks 1-3	Week 4	Day 28
Intermittent access in a two-bottle choice model (15% alcohol vs. water) with 24-h access every other day	1-week abstinence<br>5 daily aticaprant with naltrexone	ADE test
